# resPAINT: Accelerating Volumetric Super‐Resolution Localisation Microscopy by Active Control of Probe Emission**

**DOI:** 10.1002/anie.202206919

**Published:** 2022-08-23

**Authors:** Edward W. Sanders, Alexander R. Carr, Ezra Bruggeman, Markus Körbel, Sarah I. Benaissa, Robert F. Donat, Ana M. Santos, James McColl, Kevin O'Holleran, David Klenerman, Simon J. Davis, Steven F. Lee, Aleks Ponjavic

**Affiliations:** ^1^ Yusuf Hamied Department of Chemistry University of Cambridge Cambridge CB2 1EW UK; ^2^ Radcliffe Department of Medicine and United Kingdom Medical Research Council Human Immunology Unit John Radcliffe Hospital University of Oxford Oxford OX3 9DS UK; ^3^ Cambridge Advanced Imaging Centre University of Cambridge Cambridge CB2 3DY UK; ^4^ School of Physics and Astronomy University of Leeds Woodhouse Lane Leeds LS2 9JT UK; ^5^ School of Food Science and Nutrition University of Leeds Woodhouse Lane Leeds LS2 9JT UK

**Keywords:** Biophysics, Localisation Microscopy, PAINT, Single-Molecule Imaging, Super-Resolution Microscopy

## Abstract

Points for accumulation in nanoscale topography (PAINT) allows practically unlimited measurements in localisation microscopy but is limited by background fluorescence at high probe concentrations, especially in volumetric imaging. We present reservoir‐PAINT (resPAINT), which combines PAINT and active control of probe photophysics. In resPAINT, an activatable probe “reservoir” accumulates on target, enabling a 50‐fold increase in localisation rate versus conventional PAINT, without compromising contrast. By combining resPAINT with large depth‐of‐field microscopy, we demonstrate super‐resolution imaging of entire cell surfaces. We generalise the approach by implementing various switching strategies and 3D imaging techniques. Finally, we use resPAINT with a Fab to image membrane proteins, extending the operating regime of PAINT to include a wider range of biological interactions.

## Graphical abstract

A new super‐resolution technique for localisation microscopy, which combines active control of probe photophysics with stochastic binding is reported. resPAINT yields an up to 50‐fold improvement in localisation rate vs. PAINT without compromising contrast and is fully compatible with large depth of field imaging techniques. This opens the door to larger scale 3D localisation microscopy as imaging that normally takes days can now be completed in hours.

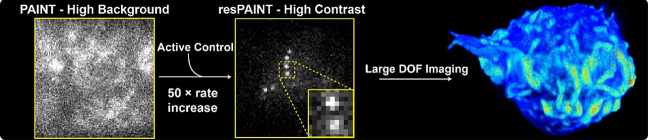

## Introduction

Single‐molecule localisation microscopy (SMLM) enables routine imaging of biological structures down to a spatial resolution of tens of nanometres.^[1]^ Fundamentally, biology occurs in 3D and therefore there has been an increasing focus on methods that can interrogate biological phenomena over increasingly larger volumes. This has motivated the development of large depth‐of‐field (DOF) SMLM.[^2^, ^3^, ^4^, ^5^] In SMLM, it becomes necessary to collect large numbers of localisations to represent increasing volumes, which is hindered by the limited speed of acquisition,^[6]^ high background and photobleaching.^[7]^ Developing techniques to overcome these limitations would offer valuable insight into numerous biological questions, including the spatial distribution of biomolecules (e.g. T‐cell activation^[8]^ and chromatin organisation^[9]^) and the study of protein‐protein interactions.[^10^, ^11^] To achieve this, suitable labelling approaches would: 1) minimise fluorescence background and 2) accommodate high emitter densities.

**Figure 1:**
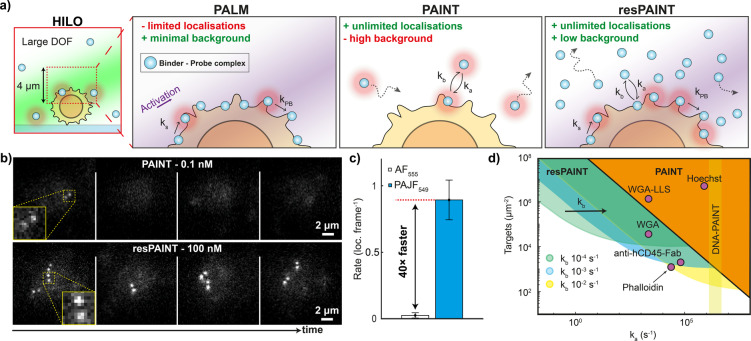
resPAINT greatly enhances localisation rates for PAINT. a) HILO: 3D SMLM is often performed using highly inclined and laminated optical sheet (HILO) excitation as this allows some optical sectioning. When combined with the DHPSF this enables imaging of a large DOF of up to 4 μm. PALM: PALM uses photoactivation of bound probes for SMLM, which achieves high contrast but finite localisation numbers. PAINT: In PAINT, probes transiently bind to targets, achieving unlimited localisations, although with increased background. resPAINT: With resPAINT, we combine active control of probe emission with PAINT to achieve practically unlimited localisations and high contrast. As probes are non‐fluorescent, much higher concentrations can be used. This concentrates probes on target that can be activated and photobleached to improve localisation rates without increasing the background (*k*_a_—association constant, *k*_b_—dissociation constant, *k*_s_—probe switching constant, *k*_PB_—photobleaching constant). b) Representative SMLM time‐series taken on fixed T cells using PAINT (WGA‐AF555, 0.1 nM) and resPAINT (WGA‐PAJF549, 100 nM) showing the increase in rate for similar backgrounds. In the DHPSF a point source appears as a pair of lobes, where the angle between the lobes represents depth. c) Quantification of (b), rates were averaged over 1000 frames and *n*=5 cells for each condition. Error bars indicate s.d. d) Operational regimes of PAINT and resPAINT with DHPSF imaging for a variety of targets (see Supporting Information Note 1 for details).

Photoactivation localisation microscopy (PALM,[^12^, ^13^] Figure 1a) and direct stochastic optical reconstruction microscopy (dSTORM[^14^, ^15^, ^16^]) have been used extensively to image biological structures in cells, but suffer from irreversible photobleaching. This is problematic for volumetric imaging, where the axial dimension increases the localisation density required for sufficient sampling.^[6]^ Probes can be refreshed, but this requires microfluidic approaches.^[17]^ PAINT (Figure 1a) circumvents photobleaching by intermittent binding of fluorescent probes to targets, enabling practically unlimited acquisition times.^[18]^ There are generally two strategies employed: in PAINT, a fluorescent binder (e.g. antibody fragments (Fabs), peptides, antigens, small molecules) intermittently attaches to the target of interest, but these are often limited by binding kinetics.[^19^, ^20^, ^21^, ^22^, ^23^] A second approach, DNA‐PAINT, involves attaching a DNA docking strand to the target, which facilitates PAINT using transiently binding complementary single‐stranded DNA probes in solution.[^24^, ^25^]

A major advantage of DNA‐PAINT is the ability to tune binding kinetics for optimised SMLM. Despite this, large‐DOF 3D imaging remains a significant challenge due to increased background from fluorescent probes in solution. Methods have been developed where probes “light up” or are concentrated on target.[^26^, ^27^, ^28^, ^29^, ^30^] While these strategies improve volumetric imaging, they retain inherent drawbacks including: unspecific binding,^[31]^ photocleavage^[32]^ and an inability to observe direct binding events (e.g. protein/protein interactions). Applying similar strategies to Fab or small molecule binders would greatly increase the range of biological interactions that can be observed using PAINT (e.g. actin‐staining using phalloidin).^[33]^

In PAINT, the background scales with probe concentration, while the localisation rate^[34]^ scales with probe concentration, binder association rate and number of binding sites (Supporting Information Note 1). Since the association rate constant is fixed, the localisation rate cannot be increased without increasing concentration, which inevitably leads to unacceptable background levels. If the target density is high then proteins and small molecule binders may achieve suitable localisation rates.[^6^, ^18^, ^20^, ^35^, ^36^, ^37^] However, in cases of molecular sparsity, as for many membrane proteins, the localisation rate is often unsuitable.

To solve this issue, we introduce reservoir‐PAINT (resPAINT, Figure 1a), which uses active control of probe emission. By ensuring that most of the probes remain non‐fluorescent, the concentration can be raised by orders of magnitude without increasing the background. This results in artificial concentration of the probe on target to create a “reservoir” of bound, non‐fluorescent probes that can be activated, and importantly, replenished. A related approach, termed PhotoActivation, Diffusion and Excitation (PhADE) microscopy has previously utilised sequential photoactivation, binder diffusion and post‐diffusion imaging to improve contrast in SMLM,^[38]^ although with limited imaging speed, corroborating the viability of our approach. Interestingly, there is also some suggestion that resPAINT may have been utilised in several studies, without formalising or fully realising the concept (referred to as no‐wash labelling).[^33^, ^39^, ^40^, ^41^, ^42^]

We demonstrate that resPAINT can improve the localisation rate for a binder by up to 50‐fold, which we combine with the DHPSF[^2^, ^3^] to perform high‐fidelity 3D imaging of the T‐cell membrane. Furthermore, we show that resPAINT is a generalisable principle that works across a variety of activation mechanisms as well as multiple large‐DOF imaging modalities. Finally, we demonstrate PAINT membrane‐protein imaging using a Fab. The ability to enhance the localisation rate without increasing background greatly extends the application range of PAINT, improving accessibility to volumetric imaging and enables super‐resolution imaging of a greater variety of biologically relevant protein‐protein interactions.

## Results and Discussion

### resPAINT Enhances the Localisation Rate of Protein Binders

We first evaluated a standard PAINT probe‐binder complex without active control of emission. We used Alexa Fluor 555 (AF_555_), covalently linked to the binder wheat germ agglutinin (WGA) that binds to the large number of N‐glycosyl sites on the cell membrane.^[6]^ We imaged the apical surface of fixed Jurkat T cells using the DHPSF (Figure 1b, 30 ms exposure, ≈10 kW cm^−2^ power density) and systematically varied binder concentration to determine the maximum (0.1 nM) that maintained an acceptable background (Supporting Information Note 1). Under these conditions, the localisation rate was prohibitive (0.02 loc. frame^−1^, Figure 1c) and would require ≈50 hours to acquire a dataset with 100 000 localisations to approach Nyquist sampling (50 nm resolution, ≈1000 loc. μm^−2^, 2D membrane imaged in 3D).

Next, we investigated the performance of a photoactivatable (PA) Janelia Fluor probe (PAJF_549_)^[43]^ attached to WGA for resPAINT. This approach requires tuning both binder concentration and the photoswitching kinetics, such that a “reservoir” of photoactivable probes is established on the membrane. Conceptually, if the binder concentration or photoactivation rate is low, the localisation rate would be impractical for super‐resolution imaging. Conversely, if the concentration or photoactivation rate is excessive, the background would decrease the signal‐to‐noise ratio. We imaged fixed T cells stained with WGA‐PAJF_549_ under identical conditions to that of WGA‐AF_555_ but now with photoactivation (0.6 W cm^−2^ power density) and higher probe concentration (100 nM, 1000‐fold larger, Figure 1b). We observed a 40‐fold improvement in localisation rate (0.85 loc. frame^−1^) for comparable background levels compared to WGA‐AF_555_ (Figure 1c, Supporting Information Figure 1, Supporting Information Movie 1). This demonstrates how replacement of a PAINT probe with a photoactivatable probe can greatly improve the localisation rate. The precision achieved is appropriate for SMLM (18 nm lateral, 38 nm axial, Supporting Information Figure 2a) and the constant localisation rate (Supporting Information Figure 3 and 4) confirmed that the probe‐binder complex was undergoing PAINT.

To explore the experimental regimes of resPAINT, we modelled the photophysical and binding kinetics of activatable probe‐binder complexes binding to targets on a cell (Supporting Information Note 1). We considered a binder with association/dissociation rate constants *k*_a_ and *k*_b_, binding to a target at a given concentration. The fluorescent binder can be activated and photobleached with rate constants *k*_s_ and *k*_PB_ respectively. This allowed us to determine the effective localisation rate and background for PAINT and resPAINT under a variety of experimental conditions. We evaluated typical ranges of these parameters to estimate the operating regimes of PAINT and resPAINT using the DHPSF (Figure 1d, Supporting Information Note 1). We found that resPAINT greatly extends the parameter ranges to a regime where it becomes possible to use low‐affinity ligands to observe biomolecules of interest (Figure 1d). The suitable range of *k*_b_ spanned approximately 10^−2^–10^−4^ s^−1^, covering a variety of biologically relevant interactions.[^44^, ^45^, ^46^] Suitable switching rate constants, *k*_s_, span a virtually identical range of 10^−2^–10^−4^ s^−1^. These data show that at lower target densities, it is necessary to have a faster dissociation rate, although it should be noted that *k*_b_ can be tuned for a given binder.^[47]^ Notable examples that become accessible with resPAINT are membrane‐protein imaging using a Fab and actin imaging using Phalloidin. While we have focused on DHPSF imaging here, resPAINT could be applied to any SMLM modality, such as total internal reflectance fluorescence (TIRF) or confocal‐PAINT microscopy and would also be applicable to astigmatic 3D‐SMLM.^[48]^ Importantly, any PAINT application using standard fluorophores could potentially be improved by changing to an activatable probe as in resPAINT.

### Whole‐Cell Volumetric Super‐Resolution Imaging Using resPAINT

**Figure 2:**
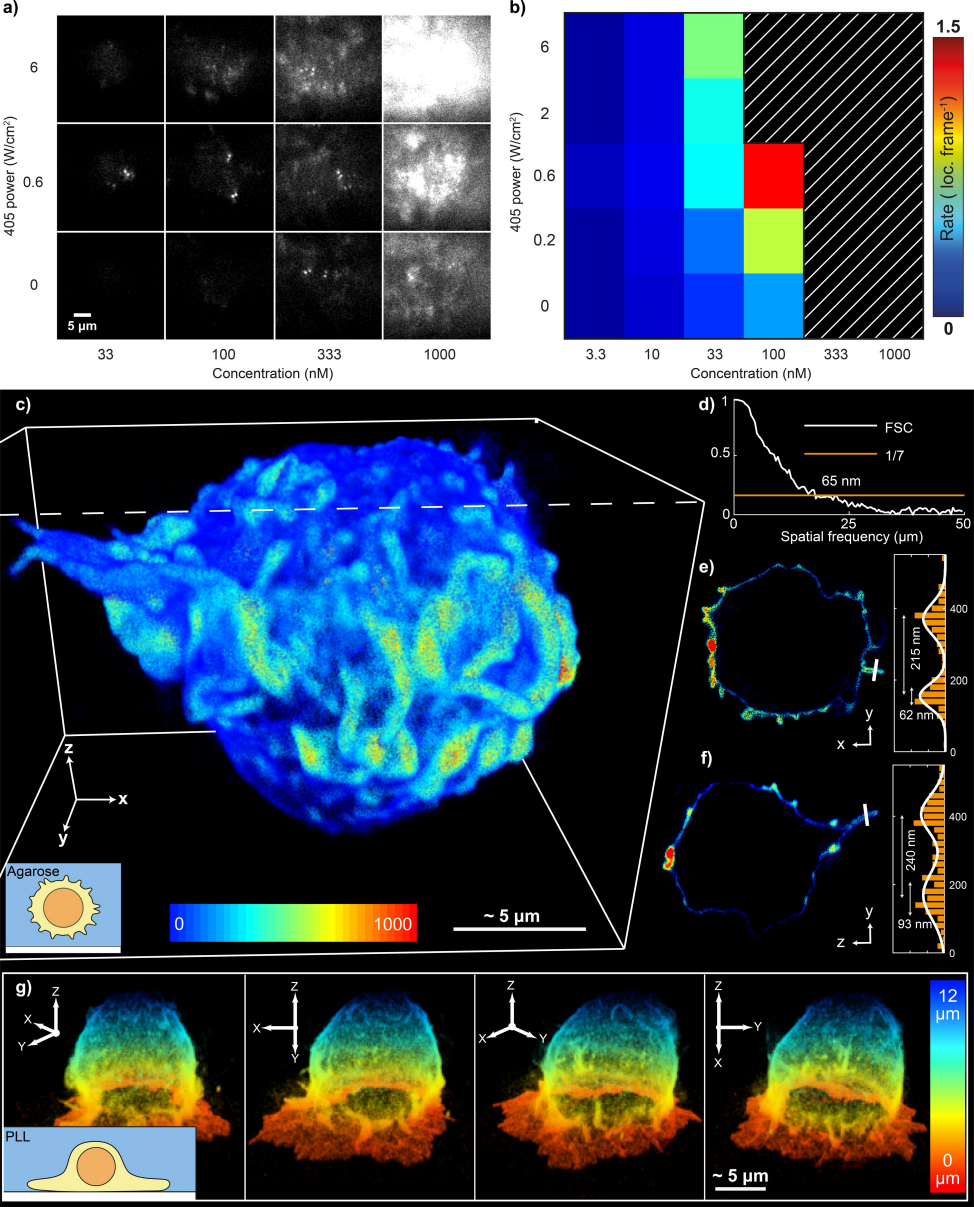
resPAINT for whole‐cell 3D super‐resolution imaging of the cell membrane. a) Representative resPAINT imaging of fixed T cells, using varying photoactivation powers and probe concentrations. b) Quantification of localisation rate in (a), highlighting identified optimal conditions (100 nM, 0.6 W cm^−2^ activation power). *n*=5 cells for each condition. The hatched region indicates the area above threshold (background>92 photons). c) 3D super‐resolution image of the membrane of a T cell acquired using resPAINT with PAJF_549_ and DHPSF. The image comprises 1 400 000 localisations, collected over 200 000 frames per z‐slice at 30 ms exposure time, and was stitched together using four 4 μm z‐slices (3.5 μm steps). The colour represents localisation density within 200 nm radius. The inset cartoon shows the T cell suspended in agarose gel. d) Fourier shell correlation (FSC)^[49]^ estimated isotropic resolution in (c) as 65 nm (1/7 cutoff). e) A y‐x slice of (c) and corresponding line plot (highlighted in white) through the microvilli (width=240 nm). f) As (e) for a y‐z slice (microvilli width=215 nm). g) As in (c), coloured by height, showing 4 rotated views of a T cell that has interacted with a PLL‐coated coverslip. Inset shows a cartoon of surface interactions.

To enable facile whole‐cell volumetric super‐resolution imaging of the cell surface we optimised the conditions of our WGA‐PAJF_549_ binder‐probe complex. The conditions shown in Figure 1b, c (100 nM and ≈0.6 W cm^−2^ 405 nm power density) were determined by finding the optimal concentration and photoactivation rate for PAJF_549_ (Figure 2a, b, Supporting Movie 2). Note that these conditions will vary depending on the binder, fluorophore and imaging modality. However, when applying resPAINT with PAJF_549_ to a different system, these conditions should provide a starting point for protocol optimisation.

We have previously used the DHPSF to image proteins on T cells using PA fusion proteins^[50]^ and dSTORM.^[8]^ However, the achievable localisation density was limited by the target density on cells as well as photobleaching. We used resPAINT to image T cells fixed in suspension and dispersed into an agarose hydrogel containing fiducial markers (for drift correction and slice alignment). Cells were imaged over four axial optical slices (4 μm DOF, 3.5 μm steps) to cover the cell volume. 800 000 frames (30 ms exposure) were acquired to measure 1 400 000 localisations that were used to construct a super‐resolution image of the whole T‐cell surface (Figure 2c, Supporting Movie 3). Fourier shell correlation (FSC^[51]^) reported a resolution of 65 nm (Figure 2d), which compared well to lattice light‐sheet PAINT^[6]^ WGA imaging (FSC resolution of 110 nm). We also assessed the ability of membrane resPAINT to resolve complex morphological features. Line profiles were applied through “finger‐like” structures to assess their resolution‐limited FWHM and membrane thickness (Figure 2e, Figure 2f), which were found to be 200–250 nm and 60–95 nm respectively, in agreement with expectations.^[52]^

We also imaged a T cell that had been allowed to interact with a poly‐L‐lysine (PLL)‐coated coverslip for ten minutes after which it was fixed (Figure 2g, Supporting Movie 4). This coating has been found to activate T cells and induce large‐scale morphology changes of the membrane.[^53^, ^54^] resPAINT was able to capture the formation of a “skirt‐like” structure (218 nm FWHM thickness, Supporting Information Figure 5) at the glass‐cell interface, demonstrating the dramatic effect of PLL on T cell‐surface interactions. In both experiments, we observed a constant localisation rate over extended timeframes (≈7 hours), demonstrating resistance to out‐of‐focus photobleaching (Supporting Information Figure 4a).

### resPAINT Is Compatible with Multiple Activation Modes

**Figure 3:**
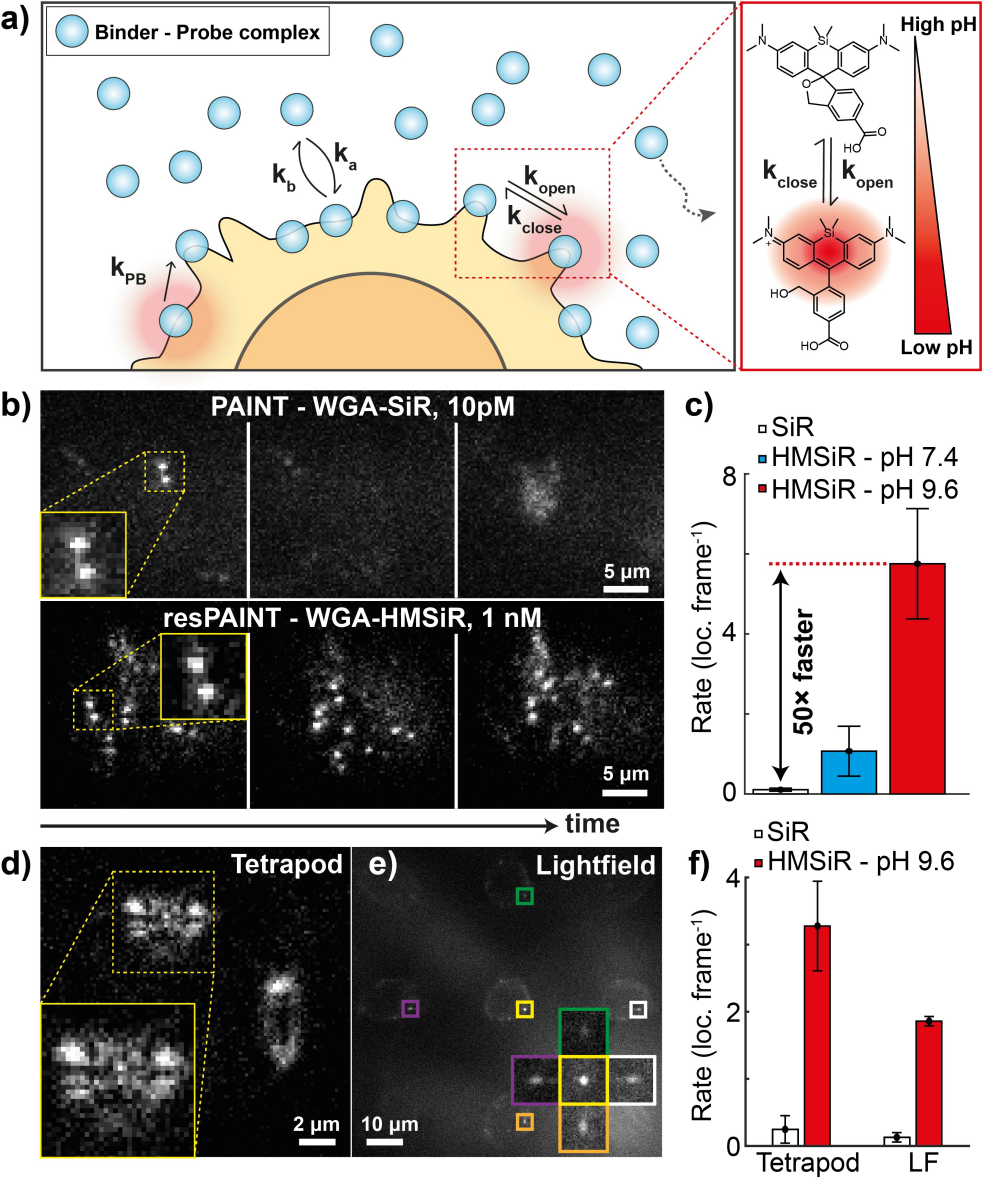
resPAINT using a spontaneously blinking probe. a) Left: Cartoon demonstrating resPAINT using a spontaneously blinking probe. Right: Schematic showing pH dependency of *K*_cyc_. b) Representative SMLM time‐series. Top: Fixed T cell imaged with PAINT (WGA‐SiR, 10 pM). Bottom: Another fixed T cell imaged with resPAINT (WGA‐HMSiR, 1 nM, pH 9.6). Display contrast was adjusted individually for each condition to aid interpretation. c) Quantification of localisation rate under background‐matched conditions. d) Representative resPAINT images taken on fixed T cells using the tetrapod PSF. e) Representative resPAINT images taken on fixed T cells using single‐molecule light‐field microscopy, inset shows one molecule viewed from 5 angles. f) Quantification of (d), (e) showing the improvement in localisation rate afforded by resPAINT. *n*=5 cells for each condition. Error bars indicate s.d.

Next, we investigated the alternative activation mechanism of spontaneously blinking probes (Figure 3a).[^55^, ^56^, ^57^] This was a good approach because: 1) no cytotoxic 405 nm requirement; and 2) fine control of off‐switching rate. We used the probe HMSiR^[55]^ that undergoes fluorescence intermittency via an intramolecular spirocyclisation reaction (Figure 3a). The cyclisation equilibrium, *K*_cyc_ *=k*_open_/*k*_close_, is affected by pH, which facilitates control of the ring opening, *k*_open_, and ring closing, *k*_close_, reactions. We therefore hypothesised that pH could be used to optimise conditions for resPAINT, by shifting the equilibrium, *K*_cyc_.^[55]^ Given a slow dissociation rate, the duty cycle of the probe is now dominated by spontaneous ring closing rather than photobleaching rate, affording greater compatibility across a range of excitation powers and exposure times.

We applied spontaneously blinking resPAINT with WGA to fixed T cells. We first compared the performance of WGA‐HMSiR to the PAINT probe WGA‐SiR and observed a modest improvement (10‐fold) in localisation rate in PBS buffer (pH 7.4). We found that this pH was suboptimal owing to an inefficient *k*_close_ (245 ms off‐time^[55]^) that was unsuitable for fast imaging (20–100 Hz), and the limited on‐off ratio limited accumulation of a probe reservoir. When we increased the pH using a sodium carbonate buffer (pH 9.6, 17 ms off‐time^[55]^), we observed a dramatic improvement (WGA‐HMSiR: 5.8 loc. frame^−1^, WGA‐SiR: 0.1 loc. frame^−1^, 50‐fold increase) for similar backgrounds (Figure 3b, c, Supporting Information Movie 5). This localisation rate resulted in overlapping fluorophores, and we determined an optimal concentration for WGA‐HMSiR DHPSF imaging (330 pM, 1.77 loc. frame^−1^) that was stable over one hour (Supporting Information Figure 4b, Supporting Information Movie 6), achieving localisation precisions of 22 nm laterally and 50 nm axially (Supporting Information Figure 2b). Increasing the pH further would limit the number of photons collected as the blinking duration would be shorter than the exposure time (Supporting Information Movie 7). The optimised pH for resPAINT imaging with HM‐SiR was then applied to T‐cell membrane imaging, as we did previously with WGA‐PAJF_549_. DHPSF resPAINT clearly resolves finger‐like microvilli structures (Supporting Information Figure 6) and their thickness compares well with expectations from scanning electron micrographs (50 nm radius^[52]^).

Next, we investigated the performance of resPAINT with alternative extended‐DOF techniques. We imaged fixed T cells using the tetrapod PSF (10 μm DOF,^[58]^ Figure 3d) and the recently developed SMLFM (5 μm DOF,^[5]^ Figure 3e). We observed improvements in the localisation rate (tetrapod PSF: 13‐fold, Supporting Information Movie 8, SMLFM: 14‐fold, Supporting Information Movie 9), which was lower than for the DHPSF due to overlapping PSFs (Figure 3f). Appropriate labelling densities for these methods (Figure 3d, e) are given in the Supporting Information (Supporting Information Movies 10 and 11).

### resPAINT Imaging Using a Fab

**Figure 4:**
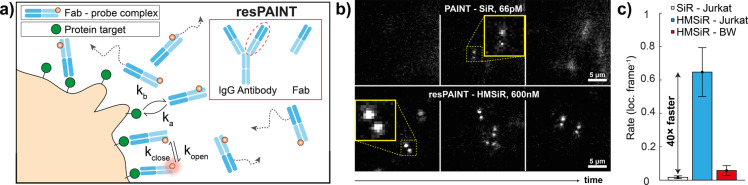
resPAINT with a Fab. a) Cartoon of resPAINT with a Fab. Inset: schematic of Fab cleaved from antibody that has suitable kinetics for resPAINT. b) Representative SMLM time‐series with fixed T cells using conventional PAINT (Fab‐SiR, 66 pM) and resPAINT (Fab‐HMSiR, 600 nM, pH 9.6). c) Quantification of localisation rate as for PAINT, resPAINT and a mouse cell control. *n*=5 cells for each condition. Error bars indicate s.d.

Having optimised and applied resPAINT to cell‐membrane imaging, we evaluated the technique in a more challenging scenario, i.e., using a Fab to image a membrane protein using PAINT. Conventionally, imaging of low‐density targets necessitates the use of DNA‐PAINT (Figure 1d) or chemical alteration of Fab off‐rates.^[47]^ DNA‐PAINT typically requires separate imaging and docking strands to form a PAINT pair, which precludes imaging of protein‐protein interactions. Conversely, resPAINT can observe interactions directly (Figure 4a). We labelled an anti‐hCD45Fab (hereafter referred to as “Fab”) with either HMSiR or SiR. We then investigated the binding of Fab to protein tyrosine phosphatase CD45.^[59]^

We have previously imaged CD45 on T cells in 3D using dSTORM and the DHPSF^[8]^ and we therefore compared PAINT (66 pM Fab‐SiR) with resPAINT (600 nM Fab‐HMSiR, Figure 4b, Supporting Information Movie 12). We first measured the dissociation rate constant of the Fab bound to fixed T cells, (pH 7.4: 1.31×10^−3^ s^−1^, pH 9.6: 1.65×10^−3^ s^−1^, Supporting Information Figure 7). These values agree with surface plasmon resonance measurements (1.59×10^−3^ s^−1^, Supporting Information Figure 8) and lie within our defined operational regime (Figure 1d). resPAINT again improved the localisation rate 40‐fold (0.61 loc. frame^−1^, Figure 4c) with localisation precisions of 22 nm laterally and 56 nm axially (Supporting Information Figure 2c). To confirm the specificity of the Fab binding to human CD45, we used a murine CD45 control cell line, to which the Fab lacks cross‐reactivity, and observed a minimal number of localisation events (0.05 loc. frame^−1^, <9 % unspecific binding, Supporting Information Movie 12). These results demonstrate that resPAINT increases the accessible range of binder kinetics beyond conventional PAINT.

### Properties of resPAINT

resPAINT dramatically improves localisation rates in PAINT without compromising contrast. This is particularly useful for large DOF volumetric imaging, as the technique facilitates acquisition of high localisation densities. The enhancements observed in this work should apply universally, including to more conventional 2D and astigmatism‐based 3D SMLM measurements, provided the following conditions are met: 1) target density ranges between 10^3^–10^8^ μm^−2^ to support reservoir accumulation and 2) binder *k*_b_ permits reservoir build up. PAINT is also limited by these factors, but resPAINT extends the range of viable binders and target densities. This makes it possible to study low abundance targets and alleviates the necessity for high *k*_a_, such that commonly used binders including Fabs, Hoechst and phalloidin, as well as low affinity antibodies,^[60]^ become accessible with PAINT (Figure 1d). resPAINT relies on build‐up of binders (*k*_a_), exchange of binders (*k*_b_) and switching of fluorophores on targets (*k*_s_). This needs to facilitate (see Supporting Information Note 1 for further details and discussion) use of higher concentration by limiting activation (*k*_s_<10^−2^ s^−1^) but sufficiently fast activation (*k*_s_>10^−4^ s^−1^) to achieve suitable localisation rates. Meanwhile, *k*_b_ must be slow (*k*_b_<10^−2^ s^−1^) to allow build‐up, but fast enough (*k*_b_>10^−4^ s^−1^) to ensure exchange before fluorophores are depleted in the reservoir. These values provide a good starting point for resPAINT under challenging conditions, but most applications would require an optimisation step as we demonstrated in Figure 2.

resPAINT limitations include needing to tune the localisation rate via two independent control mechanisms (concentration and switching). While some aspects of optimisation are specific to the probe and switching mechanism being used, we demonstrate that activation rates can be controlled via laser power (PAJF_549_) or pH (HMSiR). Importantly, the optimisations performed for these probes would apply to any system in which they are used. Therefore, resPAINT can be applied immediately in other PAINT systems as well as with other probe‐target complexes (e.g. photoactivatable,[^43^, ^61^] spontaneously blinking,[^55^, ^56^, ^57^] or fluorescent proteins^[62]^).

Aspects of the resPAINT principle have been partially explored in previous studies. PhADE^[38]^ improves contrast in SMLM by repeated labelling of targets with photoactivatable probes. While this pseudo‐PALM/PAINT approach greatly improved contrast, the sequential nature only supported slow imaging (2 Hz), whereas resPAINT is bound instead by the camera speed and photon budget. The combination of photoactivatable probes and collisional flux has also improved super‐resolution imaging in materials science with interface PAINT (iPAINT).^[63]^ However, this study had no bioimaging application, nor did it provide any kinetic framework. Within bioimaging, there is evidence for using simultaneous switching and collisional flux, although these studies also lack formal descriptions, or indeed may have applied the concept unknowingly, sometimes referred to as no‐wash protocols.[^33^, ^39^, ^40^, ^41^, ^42^] We provide the first detailed description of the kinetic requirements of resPAINT and explore the space over which it is useful for bioimaging. We generalise this concept by using a selection of probes (photoactivation and spontaneously blinking), various imaging modalities (DHPSF, tetrapod PSF, SMLFM) and apply the technique to multiple systems (whole‐cell, membrane topography and membrane proteins).

The most comparable technique to resPAINT would be DNA‐PAINT, where the rapid binding kinetics of DNA strands make DNA‐PAINT ubiquitous within SMLM, due to the high localisation precision and compatibility with low target densities. Recent modifications enhance contrast and improve localisation rates similarly to resPAINT, by adopting “light‐up” strategies.[^24^, ^26^, ^27^, ^28^, ^29^, ^30^] Furthermore, the use of left‐handed DNA has improved specificity in DNA containing samples.^[31]^ When compared, resPAINT offers some advantages in that: 1) it is compatible with DNA containing samples; 2) DNA conjugation can be complex and 3) DNA‐PAINT suffers from binding‐site depletion, although this can be somewhat mitigated.^[32]^ Indeed, a potential application of resPAINT would be imaging DNA in whole nuclei with Hoechst. This was achieved in 3D in a stimulated emission depletion (STED) PAINT mode.^[64]^ Hoechst‐HMSiR has previously been used in 2D super‐resolution imaging,^[39]^ although the authors argued against operating in PAINT mode. As they were using high‐concentration no‐wash labelling, we suggest that they may have inadvertently been applying the resPAINT principle.

While we have suggested potential binders here, suitable binding kinetics for resPAINT are typically selected against in the functional characterisation step during traditional monoclonal antibody production.^[60]^ More recently, screening for weakly binding but specific antibodies has been applied for direct PAINT of cellular targets.^[65]^ The use of Fabs with PAINT (Fab‐PAINT) has also been achieved using specialised buffers to tune binding kinetics, but this required TIRF sectioning.^[47]^ Combining Fab‐PAINT or IRIS with resPAINT would greatly relax the constraints of these approaches by improving contrast, which will facilitate large DOF volumetric imaging.

## Conclusion

We have demonstrated how resPAINT achieves an up to 50‐fold improvement in contrast or localisation rate by concentrating probes on target. This in turn extends the operational regime of conventional PAINT and facilitates volumetric 3D super‐resolution imaging using large‐DOF techniques. By simply switching to a probe with active control, it becomes possible to improve existing implementations of PAINT, if there is an excess of targets that benefits from an increase in the effective concentration. We hope that resPAINT will simplify and enable future volumetric SMLM applications in previously inaccessible areas that could include: actin phalloidin PAINT,^[33]^ intracellular LIVE‐PAINT,[^22^, ^23^] pPAINT with signalling proteins^[10]^ and IRIS with peptide fragments or antibodies.[^35^, ^65^]

## Conflict of interest

The authors declare no conflict of interest.

1

## Supporting information

As a service to our authors and readers, this journal provides supporting information supplied by the authors. Such materials are peer reviewed and may be re‐organized for online delivery, but are not copy‐edited or typeset. Technical support issues arising from supporting information (other than missing files) should be addressed to the authors.

Supporting Information

Supporting Information

Supporting Information

Supporting Information

Supporting Information

Supporting Information

Supporting Information

Supporting Information

Supporting Information

Supporting Information

## Data Availability

The data that support the findings of this study are available from the corresponding author upon reasonable request.
