# Supplementary material for: resPAINT: Accelerating Volumetric Super‐Resolution Localisation Microscopy by Active Control of Probe Emission
Source: Angew Chem Int Ed Engl. 2022 Aug 23;61(42):e202206919. doi: 10.1002/anie.202206919 (PMC9804996; doi:10.1002/anie.202206919)
Supplement: Supplementary file 10 — Supporting Information [file ANIE-61-0-s007.pdf]

Angewandte Chemie
Eine Zeitschrift der Gesellschaft Deutscher Chemiker

Supporting Information

resPAINT: Accelerating Volumetric Super-Resolution Localisation Microscopy by Active Control of Probe Emission

E. W. Sanders, A. R. Carr, E. Bruggeman, M. Körbel, S. I. Benaissa, R. F. Donat, A. M. Santos, J. McColl, K. O'Holleran, D. Klenerman, S. J. Davis, S. F. Lee, A. Ponjavic**

Supplementary Information

resPAINT: Accelerating Volumetric Super-resolution Localisation Microscopy by Active Control of Probe Emission.

Edward W. Sanders[1], Alexander R. Carr[1], Ezra Bruggeman[1], Markus Körbel[1], Sarah I. Benaissa[3], Robert F. Donat[2], Ana Mafalda Santos[2], James McColl[1], Kevin O'Holleran[3], David Klenerman[1], Simon J. Davis[2], Steven F. Lee[1,*], Aleks Ponjavic[1,4,5,*]

1. Yusuf Hamied Department of Chemistry, University of Cambridge, Cambridge, CB2 1EW, UK
2. Radcliffe Department of Medicine and United Kingdom Medical Research Council Human Immunology Unit, John Radcliffe Hospital, University of Oxford, OX3 9DS Oxford, UK
3. Cambridge Advanced Imaging Centre, University of Cambridge, CB2 3DY Cambridge, UK
4. School of Physics and Astronomy, University of Leeds, Woodhouse Lane, Leeds LS2 9JT, UK
5. School of Food Science and Nutrition, University of Leeds, Woodhouse Lane, Leeds LS2 9JT, UK

* To whom correspondence may be addressed. Email: sl591@cam.ac.uk, and a.ponjavic@leeds.ac.uk.

Supplementary Materials: Methods

Cell culture: Jurkat T cells (ATCC TIB-152) were grown in RPMI (Sigma-Aldrich, Madison, WI), while mouse thymoma BW5147 cells were cultured in Joklik-modified Minimum Essential Medium (JMEM) (Sigma-Aldrich). Both culture media were supplemented with 10% fetal calf serum (FCS) (PAA), 10 mM HEPES (Sigma-Aldrich), 1 mM sodium pyruvate (Sigma-Aldrich), 2 mM L-glutamine and antibiotics [50 units penicillin, 50 µg streptomycin and 100 µg neomycin per mL] (Sigma-Aldrich). Cells were maintained at 37˚C and 5 % CO_2 during culturing and, typically, kept at a density between 5-9 × 10^5 cells mL^{-1}.

Protein Labelling: The corresponding protein WGA (L9640, Sigma-Aldrich, UK) or CD45 Fab (prepared as in previous work)[1] was added in a 1:10 molar ratio to the desired dye - $PAJF_{549}$ (Tocris Bioscience, UK) or HMSiR (Sarafluor-650B, Kishida Chemical Company, Japan) - in 0.02 µm filtered (6808-2002, Cytiva, MA) phosphate buffered saline (PBS, 10010-023, Gibco, MA). 1 M sodium bicarbonate (Sigma-Aldrich) in ultrapure water was added to achieve a 0.1 M concentration in the reaction volume and the reaction was left in the dark for 1.5 hours at room temperature. The protein-dye conjugate was purified by 3 rounds of size exclusion chromatography (Bio-Spin 6 column, BioRad, CA) and then aliquoted in 5 µL portions and stored at -80˚C until required.

Cell preparation: ~10^6 of cells were centrifuged (600 × g, 2 minutes) and the supernatant was removed before washing once with filtered PBS. The cells were fixed in 0.8 % paraformaldehyde (28906, Thermo Scientific, MA) and 0.2 % glutaraldehyde (G5882, Sigma-Aldrich) for 15 minutes at room temperature. The cells were then washed three times in filtered PBS and then resuspended in ~100 µL of filtered PBS.

Cell-coated coverslip preparation for apical surface imaging: Glass slides (24 × 50 mm borosilicate, thickness No. 1.5, VWR international, PA) were cleaned for 30 minutes with argon plasma (PDC-002, Harrick Plasma, Ithaca, NY) and then coated with poly-L-lysine (PLL, 150-300 kDa; P4832; Sigma-Aldrich) for 15 minutes. The slides were then washed three times with filtered PBS before ~ 30 µL of PBS was placed with 5-20 µL of cells in PBS and the cells were allowed to settle on the surface for 45 minutes.

T-cell coated coverslip preparation for whole cell imaging: T cells were adhered to a coverslip using PLL as before. Fiducial markers were prepared as follows. A 100 µL solution containing 50 µm agarose beads (20349, ThermoFisher) was incubated with PLL solution in a 1:1 ratio for 10 minutes followed by centrifugation at 1500 × g for 1 minute. The beads were washed three times with filtered PBS and incubated with nitrogen vacancy fluorescent nanodiamonds (798134, Sigma) in a 1:1 ratio for 10 minutes at room temperature. The labelled beads were then washed three times with filtered PBS and resuspended in 100 µL of filtered PBS. Onto a pre-prepared T-cell coated coverslip was added 3 µL of fluorescent nanodiamond-coated 50 µm diameter agarose beads, which were allowed to settle on the surface. The sample was heated to 37°C and 50 µL of 1 % agarose solution in filtered PBS was added and allowed to settle for 10 minutes. The sample was then allowed to cool to room temperature before 50 µL of filtered PBS was added to the set agarose.

DHPSF Microscopy: The microscope used for Fab and WGA imaging with the DHPSF was as in our previous work,[2] incorporating a 1.27 NA 60× water immersion objective lens (1.27 na Plan Apo VC 60×, Nikon) and a quad-band dichroic (Di01-R405/488/561/635-25x36, Semrock) with minor alterations to the

optics in the emission path. Namely, for SiR and HMSiR dyes a phase mask (PM) optimised to a different wavelength (650 nm, DoubleHelix, Boulder, CO) was used and the fluorescence signal was isolated by placement of band-pass and long-pass filters (FF02-675/67-25 and BLP01-647R-25, Semrock) immediately before the camera. Excitation light on the sample was filtered using a bandpass filter (FF01-640/14-25, Semrock) In the case of the dyes AF_{555} and $PAJF_{549}$ the PM was replaced with a 580 nm optimised PM (DoubleHelix, Boulder, CO). The fluorescence signal was isolated by use of band-pass and long-pass filters FF01-580/14-25 and BLP02-561R-25 (Semrock, Rochester, NY) and collected by an EMCCD (Evolve Delta 512, Photometrics, Tucson, AZ) operating in frame transfer mode. The excitation light was filtered by use of a bandpass filter (LL02-561-25, Semrock). The DHPSF was calibrated by use of Tetraspeck beads (Thermofisher, T7279) for both PMs and filter combinations, where the fluorescent bead slides were prepared on PLL coated coverslips as in previous work.[2]

resPAINT imaging of apical T-cell surface: The liquid was carefully removed from a T-cell coated coverslip and the surface was then gently washed with a prediluted solution of probe at the required concentration in either filtered PBS or, in the case of HMSiR, in filtered pH 9.6 sodium carbonate-bicarbonate buffer and then imaged on the custom-built DHPSF microscope. For experiments that involved HMSiR and SiR, a 20 ms exposure time was used in both the WGA and Fab imaging cases and a continuous 641 nm excitation beam at (~5 kW cm^{-2}) was used in a HILO illumination configuration. The photoactivation mode experiments were conducted with 30 ms exposure times while a continuous 561 nm excitation beam (~10 kW cm^{-2}, measured after objective) was used in combination with a continuous 405 nm beam used for activation at a range of power-densities during optimisation experiments (~ 0-6 W cm^{-2}, measured after objective). An image was collected that centred on the apical surface and contained most of the DHPSF's 4 µm depth of field. In order to quantify the resPAINT improvement, the background was matched in conventional PAINT and resPAINT cases by titrating probe into the imaging volume before an average z-projection of an area off cell was taken and the counts measured for a small ROI in the centre of the frame. The localisation rates at similar background levels were compared. The quoted improvements are indicative of the difference in localisation rates under these matched conditions.

Whole cell resPAINT imaging: A coverslip prepared for whole cell experiments was imaged using the same excitation and emission path configuration as for the optimisation $PAJF_{549}$ experiments. Continuous 561 nm illumination (~ 5 kW cm^{-2}, measured before objective) and 405 nm excitation (~ 5 W cm^{-2}, measured before objective) was incident on the sample. Four ~4 µm planes were imaged, where each position contained at least one fiducial marker shared with adjacent planes to allow alignment of localisations post-drift correction. 200,000 frames were recorded at 30 ms exposure for each plane and then the focus was shifted in 3.5 µm steps using a piezo z-stage. An auto-focus script based on the DHPSF of fiducial markers was written in Beanshell and used to maintain the axial position of the sample during acquisition of individual planes.

The resolution of resulting images was evaluated using Fourier shell correlation with a custom MATLAB script. The 3D point cloud dataset was randomly split into two equal parts. This was then used to create a 3D image using 10 nm^3 voxels, where each point contributed to a Gaussian intensity distribution with σ_{xy} = 40 nm and σ_z = 60 nm. Finally, an existing script[3] for Fourier shell correlation in MATLAB was applied to the two images to determine the resolution at the 1/7 intercept.

anti-hCD45-Fab dissociation rate imaging and rate constant calculation: T cells were prepared and adhered to a coverslip using PLL, as for apical surface imaging, before incubation with 200 nM of anti-hCD45-Fab-SiR (Gap8.3-Fab-SiR) for 15 minutes. Imaging was performed on a bespoke microscope as in previous work[4] using a 641 nm excitation laser (Obis, Coherent). The beam was filtered with an appropriate excitation bandpass filter (FF01-640/14-25, Semrock) and circularly polarised using a wavelength specific quarter-wave plate. The beam was then expanded, collimated and aligned for epifluorescence with an air immersion objective (20× Plan Fluor, NA 0.5, air immersion, Nikon Corporation) mounted on an inverted microscope body (Eclipse Ti2, Nikon Corporation). Emitted light was collected by the same objective lens and separated from excitation light by way of a dichroic mirror (Di01-R405/488/561/635, Semrock) and an appropriate emission bandpass filter (FF01-692/40-25, Semrock). The emitted light was then expanded and focused onto an electron-multiplying charge-coupled device (Evolve 512, Photometrics) for imaging, where the pixel size was 535 nm. A stack of single images was taken in 20 s intervals, with an EM gain of 250, where the exposure time was 100 ms and the power density incident on the sample was ~0.3 W cm^{-2}. The dissociation rate constant was measured by fitting the decay in fluorescence signal over time to an exponential function in Fiji.

anti-hCD45-Fab surface plasmon resonance measurements: Gap8.3-CD45 interactions were analysed on a Biacore 8k instrument (Cytiva Life Sciences) at a flow rate of 10 µl min^{-1} with HBS-P running buffer. A Protein A Chip (Cytiva Life Sciences) was used to capture Gap8.3 (~2000RU) onto Flow cell 2 (FC2) at 10 µl min^{-1}. Before injection of CD45 the chip surface was conditioned using 3 injections of HBS-P for 60 s each. Serial dilutions of CD45D1-D4 or CD45RABC were injected for 60 s at 30 µl min^{-1} over both FC1 (reference) and FC2 using single cycle kinetics with a final dissociation time of 300 s. A blank run was also performed using PBS in HBS-P to match the serial dilutions of CD45 for blank subtraction and together with FC1 used for double reference subtraction. All measurements were performed at 20°C. Results were analysed using the Biacore Evaluation Insight Software (Cytiva Life Sciences) using 1:1 kinetic model binding.

DHPSF fitting: The whole cell dataset was fitted using easyDHPSF[5] as previously described.[2] Briefly, a calibration dataset was acquired by scanning the stage in 40 nm steps. Using the calibration file, camera parameters and manually selected thresholds, easyDHPSF produced a point cloud of localisations. Drift was corrected based on individual fiducial markers present in each plane. The five planes were aligned by identifying overlapping fiducial markers between planes and correcting localisation positions. Repeated localisations were removed via temporal filters where a localisation was removed if within 500 nm and 0.5 s of a previous localisation. For the images presented and analysed in Fig. 2, a density filter with 200 nm radius was used to remove spurious noisy localisations with less than 5 neighbours.

For all other datasets, DHPSF fitting was done using a custom MATLAB script (currently available at https://github.com/TheLaueLab/DHPSFU). Image sequences were first analysed with the GDSC plugin PeakFit,[6] to extract localisations. These were paired using the DHPSFU MATLAB script, which uses a PSF calibration file to accurately assign x,y,z positions to the point pairs. Repeat localisations within 20 frames and a 200 nm radius were combined into singles using a temporal filter (~0.5 s depending on exposure time).

Tetrapod microscopy: A piezoelectric deformable mirror (DMP40-F01, Thorlabs) was used to generate a tetrapod PSF based on a previous implementation.[7] The deformable mirror was placed in the conjugate back focal plane of the objective (Plan Apo, 60 ×A/1.40 Oil, DIC H, inf/0.17 WD 0.21, Nikon) using a relay of achromatic doublet lenses (AC254-200-A, Thorlabs). The deformable mirror was controlled using the manufacturer's software (version 3.2, Deformable Mirror Software Package, Thorlabs). The tetrapod pattern was generated using a 0.25:-0.75 ratio of secondary and primary astigmatism. The microscope setup was based on a Nikon Eclipse Ti2-E. Two 638 nm diode lasers (each 180 mW, 06-MLD 638 nm, Cobolt) were focused to the back focal plane of the objective using a lens (AC254-250-A, Thorlabs) on a linear translation stage to allow HILO illumination. A dichroic (Di01-R405/488/532/635, Semrock), and emission filters (FF02-675/67-25 and BLP01-647R-25, Semrock) were used. The power density at the sample was ~2.5 $kWcm^{-2}$. A sCMOS camera was used (Prime 95B, Teledyne Photometrics) and controlled with µManager 2.0 gamma.[8] Data analysis was performed using the ImageJ plugin ZOLA-3D.[7] The experimental PSF was modelled using 66 Zernike coefficients. Localisations were filtered based on goodness of fit and photon number (>2,000 photons).

Lightfield Microscopy: A bespoke lightfield microscope was used as in previous work.[9] Jurkat T-cell membranes were imaged using WGA-SiR or HMSiR with continuous excitation at 638 nm (~1 kW cm^{-2}). HILO illumination configuration was used to minimise fluorescence background to image a plane near the apical surface of Jurkat T cells. Quantification of resPAINT improvement was conducted in the same way as for DHPSF images

Light field fitting: The microlens array in the SMLFM system encodes the 3D position of the point emitters in the displacement of the focused image from the optical axis of each lenslet. Sub-diffraction localisation of the point emitter images were performed by fitting a 2D Gaussian profile using the ThunderSTORM package.[10] The 3D localisation was estimated using the previously described method.[9] This uses knowledge of the optical model and the set of 2D localisations to estimate a 3D localisation for each point emitter. The 3D fitting parameters were: Perspective views (3-5), 2D Gaussian fitting widths (0.4 - 1.2), paraxial angle for grouping (0.5 ^{o}), 3D fit threshold (0.5 µm) and intensity threshold of (200 photons).

Technical Note 1: Photophysical kinetics of resPAINT

Consider a system where

[A]	= concentration of binder-probe complex
B_{max}	= total number of binding sites for A
B_{bound}	= number of bound sites
$B_{reservoir}$	= number of bound activatable molecules
B_{active}	= number of bound fluorescent molecules
B_{PB}	= number of bound photobleached molecules
k_a	= association rate constant of the binder
k_b	= dissociation rate constant of the binder
k_d	= equilibrium constant
k_s	= switching rate constant of the probe
k_{PB}	= photobleaching rate constant of the probe

$$B_{bound} = B_{reservoir} + B_{active} + B_{PB}$$

$$Number\ of\ vacant\ sites = B_{max} - B_{bound}$$

A binder, labeled with a blinking fluorophore, is added to an aqueous solution at a concentration, [A]. The intermittent binding to a target on cells is governed by an association rate constant, k_a, and a dissociation rate constant, k_b. The fluorophore is assumed to be in a dark state given a large on-off ratio. Thus, the concentration of target sites bound with dark activatable fluorophores, $B_{reservoir}$, depends on the association kinetics and the total available binding sites, B_{max}.[11] The dark fluorophore can switch into a fluorescent state, via photoactivation or spontaneous blinking, with a switching rate constant, k_s. Assume that the proportion of dye switching from dark to photobleached is negligible. Under these conditions, the change in the dark bound fluorophore concentration, $d(B_{reservoir})/dt$, can be described by

$$\frac{d(B_{reservoir})}{dt} = k_a[A](B_{max} - B_{bound}) - k_b B_{reservoir}$$

where B_{bound} comprises the bound activatable concentration, $B_{reservoir}$, the bound fluorescent concentration, B_{active}, and the bound photobleached concentration, B_{PB}. The fluorescent binder can dissociate with the dissociation rate constant of the binder, k_b, or it can photobleach with a rate constant, k_{PB}. Note that the on-switching rate of bound dark fluorophores, $k_s B_{reservoir}$, is the quantity that is measured in the experiment and corresponds to the localisation rate in SMLM. For spontaneously blinking probes, the off-switching rate is determined by k_{PB} and the rate of ring closing (k_{close} in Fig 3a). For simplicity, we assume that the probe does not undergo spirocyclisation multiple times, *i.e.* the photobleaching rate is dominant. Under these conditions, the change over time, *dt*, in the fluorescent binder concentration, B_{active}, can be described by

$$\frac{d(B_{active})}{dt} = k_s B_{reservoir} - k_b B_{active} - k_{PB} B_{active}$$

The photobleached binders can dissociate with the dissociation rate constant of the binder, k_b. Under these conditions, the change in photobleached binder concentration, B_{PB}, can be described by

$$\frac{d(B_{PB})}{dt} = k_{PB} B_{active} - k_b B_{PB}$$

Consider the case of WGA at a concentration [A] = 330 nM binding to a cell membrane imaged with the DHPSF. In this case, k_a = 5,300 $M^{-1}s^{-1}$, k_b = 1.2×10^{-3} s^{-1}, equilibrium constant, k_d = 230 nM, and B_{max} = 3.8×10^6, assuming a 100 μm^2 membrane area imaged with DHPSF at lectin density 3.8×10^4 μm^{-2}.[12] WGA is labeled with $PAJF_{549}$ with k_s = 10^{-3} s^{-1} and k_{PB} = 100 s^{-1}, which depend on activation and excitation power densities.

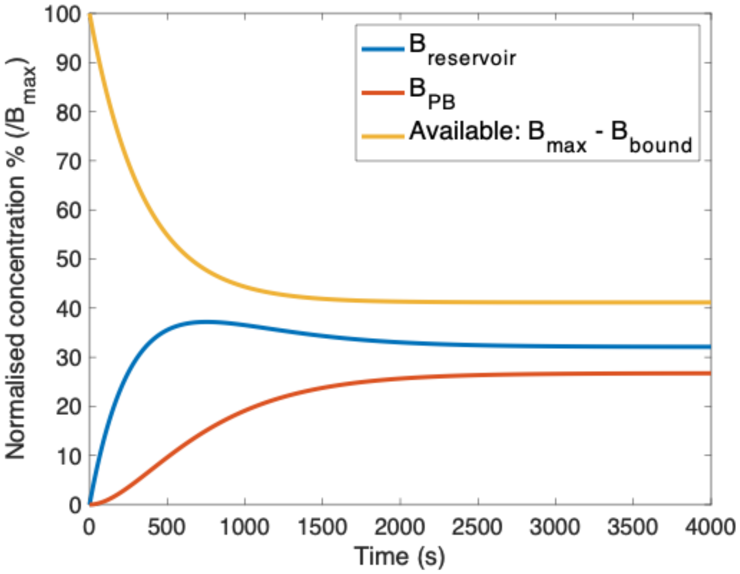

Figure A1: resPAINT kinetics. Evolution of bound reservoir, bound photobleached and unbound populations on the cell membrane over time. The reservoir, $B_{reservoir}$, approaches a steady state from which probes can be both replenished and activated for localisation imaging.

Building a reservoir. Figure A1 shows how these populations evolve over time. Initially, there is a build-up of bound dark sites that can be activated. As these switch into a fluorescent state and eventually photobleach, there is a build-up of bound photobleached sites. Due to the binding kinetics of WGA, an equilibrium is reached at around 10 minutes, where there is a constant supply of bound dark sites that can be activated for localisation microscopy. As the localisation rate is proportional to $B_{reservoir}$, the maximum is reached in approximately 10 minutes for WGA-$PAJF_{549}$.

This analysis enables comparison between conventional PAINT and resPAINT for a specific probe. Owing to the fast photobleaching rate, the background is controlled by the activation rate of probes in solution of the observable volume, V, in the experiment, given by $k_s[A]V$, while the localisation rate is given by $k_sB_{reservoir}$. In a typical DHPSF imaging volume, V, of $10\times10\times4$ μm^3, the background for WGA-$PAJF_{549}$ at 330 nM is 80 localisations while the localisation rate is 1,220 loc.s^{-1}. PAINT would have a similar background at a concentration of $[A]k_s^{-1}$ = 330 pM, due to fluorescent probes effectively instantaneously diffusing into the excitation volume. At this point the localisation rate in PAINT would be $k_a[A]B_{max}$ = 6.52 loc.s^{-1}, or rather resPAINT could reach a theoretical improvement upper limit of up to 188 times faster than PAINT in the case of WGA. Empirically we determine an increase of ~50 fold, which is likely explained by multiple assumptions made in the this analysis (*i.e.* rate constants taken from different cellular systems[12]) and the imperfect nature of the single-molecule experiments (*e.g.* diffusion out of the excitation volume, homogeneity of the excitation volume).

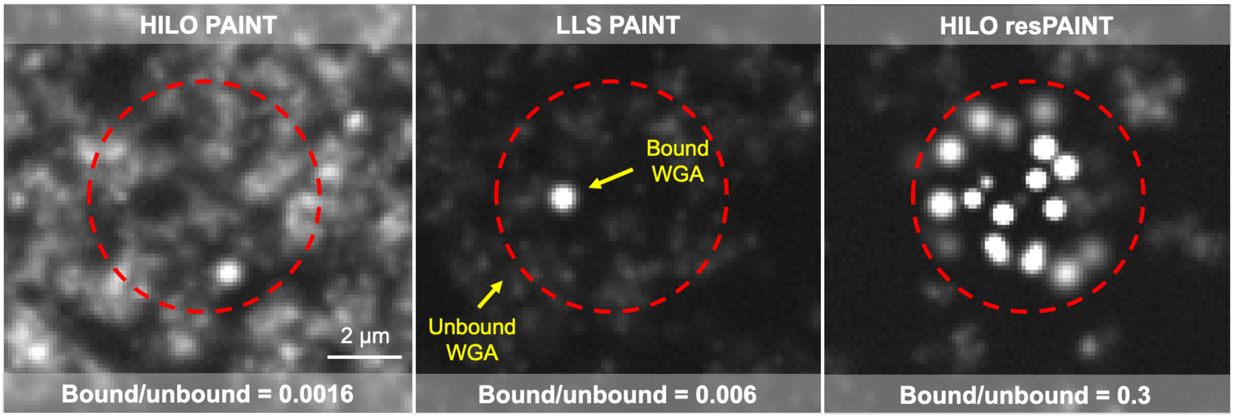

Figure A2: Simulation of an abundant target (glycocalyx). Binding and photophysical kinetics for WGA with a conventional PAINT fluorophore (1 nM) and a photoactivatable resPAINT fluorophore (100 nM, $k_s = 0.001\ s^{-1}$). The red circle indicates the cell area. The bound/unbound ratio represents the signal/background ratio. LLS improves conventional PAINT 4-fold by reducing out-of-focus excitation, whereas resPAINT provides a 180-fold improvement.

Simulations. These findings can be supported by simulating the images that would result from these binding and photophysical kinetics (as described above). We simulated the diffusion ($D = 76\ \mu m^2\ s^{-1}$) of WGA-probe complexes in solution to compare the performance of PAINT, lattice light-sheet PAINT[13] and resPAINT with similar backgrounds across all three techniques. We evaluate the quality of the images by considering the bound-active/unbound-active molecule ratio, where higher is better.

HILO PAINT with WGA produces poor image quality as there is a large excitation volume, resulting in unwanted fluorescence background caused by emissive diffusing probes. This can be ameliorated somewhat by moving to a confined excitation geometry like LLS. For WGA at 1 nM (Figure A2, Supplementary Video 13), LLS improves the image 4-fold, to give a suitable background that enables single-molecule imaging at the given localisation rate. When HILO is combined with resPAINT, the excitation volume (and therefore background) is again increased 4-5 times. Despite this, due to building up of the reservoir (through higher concentration 100 nM), resPAINT can still generate substantially, up to 180-fold, better image quality compared to PAINT.

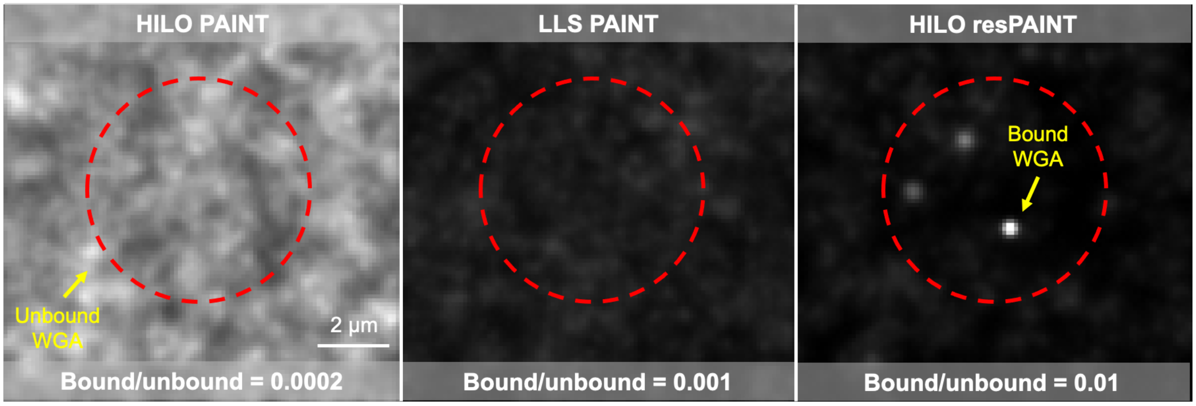

Figure A3: Simulation of sparse target case - Binding and photophysical kinetics for a Fab (30,000 targets on cell) with a conventional PAINT fluorophore (10 nM) and a photoactivatable resPAINT fluorophore (1,000 nM). resPaint becomes essentially required for imaging under these conditions.

When the target density, B_{max}, is reduced as in the case of a protein binder, like a Fab, the binder concentration needs to be greatly increased to provide a suitable localisation rate. In this case resPAINT becomes essential for imaging (Figure A3, Supplementary Video 14).

Exploring the accessible regime of resPAINT (Figure 1d). By solving the system of linear differential equations, the equilibrium concentration of $B_{reservoir}$ can be determined. This was used to create Figure 1d. The switching rate constant, k_s, and dissociation rate constant, k_b, were varied over large ranges, for a given association rate constant, k_a, and target density, B_{max}/A, where A is area. Note that the target density refers to targets within the DHPSF DOF (4 μm), projected onto an area in image space. These targets can for example be 1D (actin), 2D (membrane) or 3D (DNA), but become 2D when projected onto the image plane. If a condition was found where the localisation rate was larger than 1 $s^{-1}\mu m^{-2}$ and the background was lower than 0.6 molecules μm^{-2} (matching LLS conditions[13]) then it was coloured in Figure 1d for resPAINT and PAINT respectively.

These thresholds were selected as they agreed with our experimental conditions for WGA-PAJF$_{549}$. Therefore, they should be interpreted as a guide for imaging regimes rather than an absolute quantification. However, they should provide a good starting point for identifying whether a certain application would be feasible with resPAINT. Furthermore, the relative scaling of PAINT to resPAINT is linear such that the improvement is independent of the choice of thresholds. Practically this means that for different thresholds, the targets would remain static on Figure 1d, while the boundaries would move.

Discussion on the interplay of kinetic rates and recommendations for resPAINT experiments.

Conceptually. The dissociation rate constant needs to be slow enough to support the build-up of a reservoir, but fast enough to exchange the reservoir and avoid buildup of photobleached binders. Meanwhile the activation rate needs to be slow enough to avoid depleting the reservoir, but fast enough to achieve the required localisation rate.

Providing specific rules for optimised experiments is complicated due to the large number of variables, k_a, k_b, k_s, k_{PB}, B_{max}, as well as requirements (localisation rate and background).Therefore it is possible there are multiple degenerate solutions that would give rise to a successful resPAINT experiment. However here we can report some generalised principles that will help users wanting to implement resPAINT.

Analytically. The expression that needs to be maximised is the localisation rate ($k_s B_{reservoir}$), which at equilibrium is defined as:

$$B_{reservoir} = \frac{k_a k_b k_s [A] B_{max}}{k_b k_s + k_b^2 + k_a k_b [A] + k_a k_s [A]}$$

This typically means that k_a and B_{max} should be as high as possible, but this cannot typically be modified for a given binder.

Worked Example. Following our defined thresholds of localisation rate 1 loc μm^{-2} and background of 0.6 mol um^{-2}, we determine the maximum contrast for WGA (k_a = 5,200 M^{-1} s^{-1}, k_b = 1,200 s^{-1}, B_{max} = 3.8×10^4 um^{-2}) to be at a concentration of 62 nM and a switching rate of 1.8×10^{-4} s^{-1}. While we have not directly measured the switching rate, the concentration agrees with our empirically determined experimental conditions for WGA imaging. In the case of Fab (k_a = 10^5 $M^{-1}s^{-1}$, k_b = 10^4 s^{-1}, B_{max} = 2,000 μm^{-2}), we find an optimal concentration of 23 nM and a switching rate constant of 3.4×10^{-3} s^{-1}. These parameters would greatly depend on threshold and application, but adhere to the general ranges we have identified elsewhere.

In order for resPAINT to have an appreciable increase in effective concentration, k_s should be smaller than about 10^{-2} s^{-1} (1% per second). However, if k_s is much smaller than 10^{-4} s^{-1} then too many targets would be required to achieve a suitable localisation rate. Under limiting conditions, B_{max} will be about 50,000 and at high binder concentration this would require an switching rate constant of 10^{-3} s^{-1} to achieve 1 loc frame^{-1}

with 20 ms exposure (typical to our conditions). With 10^{-3} s^{-1} switching rate constant, most molecules will activate in 1,000 s, corresponding to the approximate time over which fluorophores replenish, suggesting a suitable dissociation rate of about 10^{-3} s^{-1}. This agrees with Figure 1d that shows that resPAINT under our experimental conditions is mostly appropriate for k_b of 10^{-2} to 10^{-4} s^{-1}. The faster the dissociation rate is, the faster the photoactivation rate should be to make use of fluorophores before they exchange.

Practically. We would suggest the following protocol as a route to optimize photoactivation resPAINT conditions.

1) Firstly perform a serial dilution of the probe-binder complex, typically beginning with a low concentration and titrating additional complex logarithmically (100 pM, 1 nM, 10 nM, 100 nM, 1 μM) until observation of either:
    i) single molecules undergoing PAINT, or
    ii) the background levels become visible to the detector.
2) Secondly, optimise the photoactivation rate. Begin to increase the extent of UV exposure, typically vary the photon fluence from an initial value of around 0.1 W cm^{-2}.
3) Measure and plot the number of localisations with time. Fit this to a linear function, where the linearity of the plot will confirm PAINT. The gradient of this graph is a reporter of the efficiency of the resPAINT system, and as such, optimisation seeks to maximise this gradient.
4) Iterate steps 1 and 2 incrementally until optimal conditions are reached (effectively creating a matrix of conditions as in Figure 2a).

N.B. In the case of spontaneous switching, only the concentration is typically varied, and so is not discussed here.

Supplementary Figures

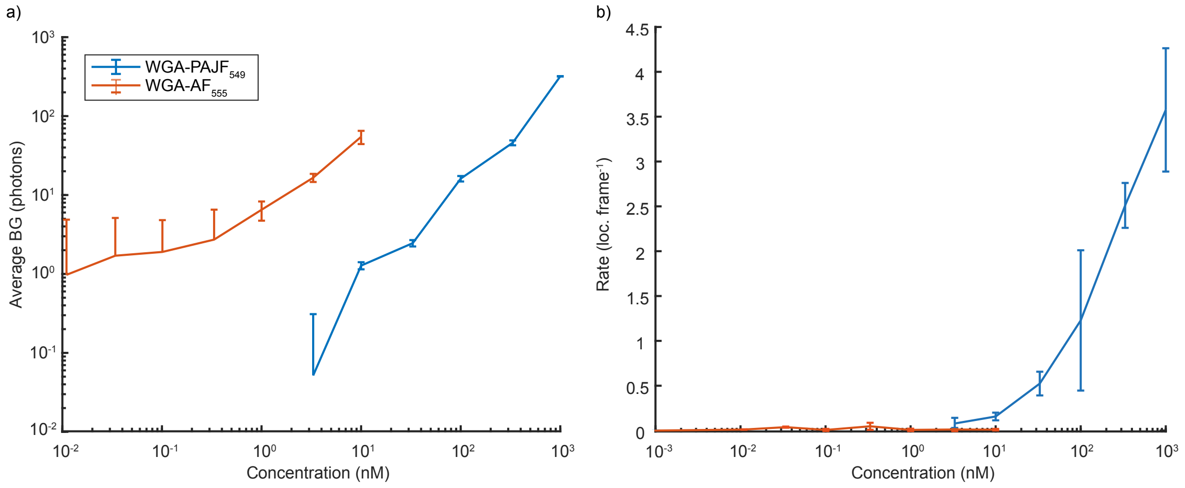

Supplementary Figure 1: Relating background fluorescence and localisation rate to concentration for both conventional PAINT and resPAINT. a) Background fluorescence as a function of concentration for both WGA-AF_{555} and WGA-$PAJF_{549}$. In both cases, background increases linearly with concentration. The data show that for comparable levels of background, WGA-$PAJF_{549}$ can support orders of magnitude larger probe concentrations due to the background suppression of resPAINT. *n* = *5* cells for each condition. Error bars indicate s.d. **b)** Localisation rate as a function of concentration for WGA-AF_{555} and WGA-$PAJF_{549}$. Owing to the ability to use far higher probe concentrations, and the formation of an inactive probe reservoir on targets, resPAINT can achieve improved localisation rates for comparable levels of background. *n* = 5 cells for each condition. Error bars indicate 1 s.d..

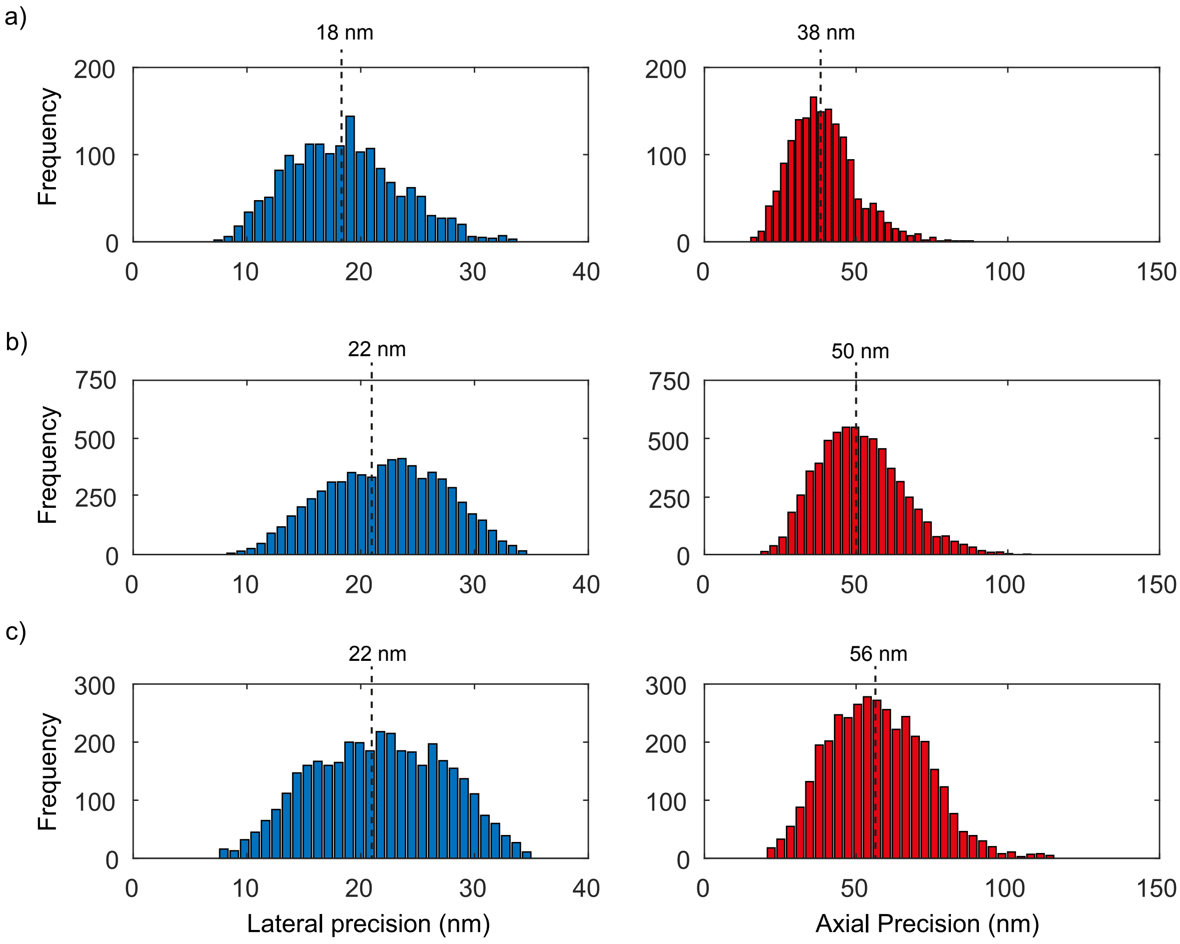

Supplementary Figure 2: Localisation precision in resPAINT imaging. Precision histogram for typical resPAINT experiments with **a)** WGA-PAJF$_{549}$ **b)** WGA-HMSiR and **c)** anti-hCD45Fab-HMSiR. We determine median lateral precisions (left) of 18 nm, 22 nm and 22 nm for WGA-PAJF$_{549}$, WGA-HMSiR and anti-hCD45Fab-HMSiR respectively. Median axial precisions (right) were also determined as 38 nm, 50 nm and 56 nm for WGA-PAJF$_{549}$, WGA-HMSiR and anti-hCD45Fab-HMSiR respectively.

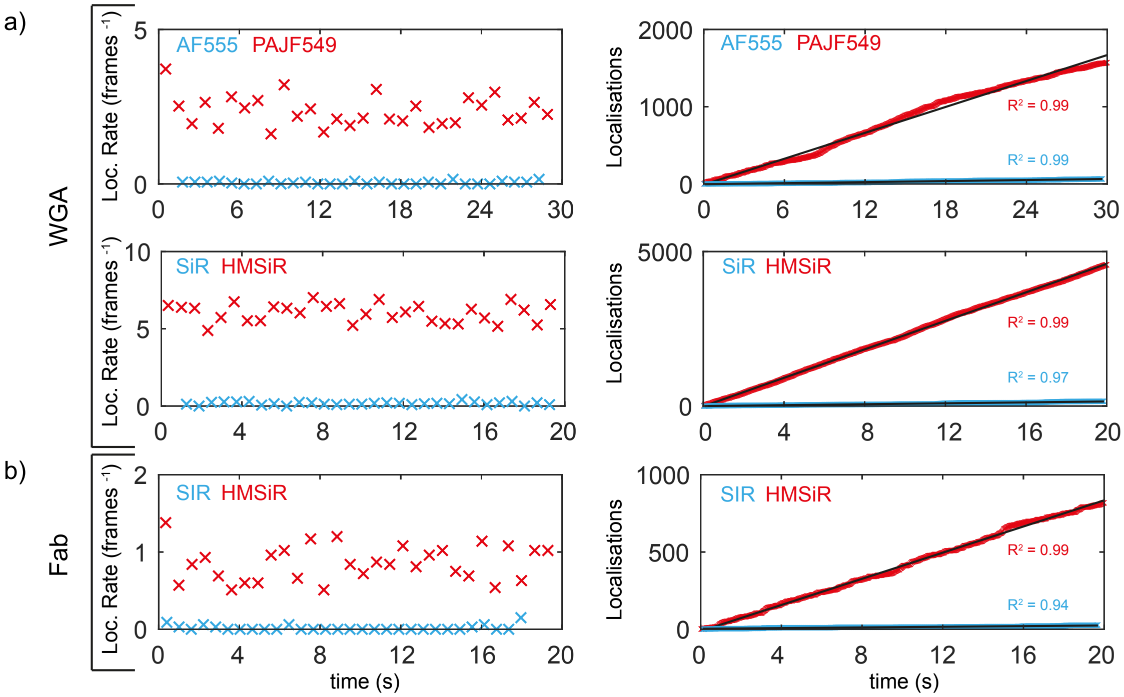

Supplementary Figure 3: **resPAINT maintains a stable localisation rate over time, indicative of a PAINT binding mode. a)** Localisation rate and cumulative localisations as a function of time for membrane imaging with WGA **Left:** Histograms of localisation rate as a function of time (bin width is 1 second for $PAJF_{549}/AF_{555}$ and 0.6 seconds for HMSiR/SiR). In both cases, the localisation rate does not change appreciably over time, with resPAINT probes affording a significantly higher localisation rate. **Right:** Cumulative localisations as a function of time, showing a linear response. **b)** As in (a), but for anti-hCD45Fab imaging of CD45 membrane protein and the corresponding conventional PAINT experiment with SiR. A constant localisation rate with time is indicative of PAINT-style imaging.

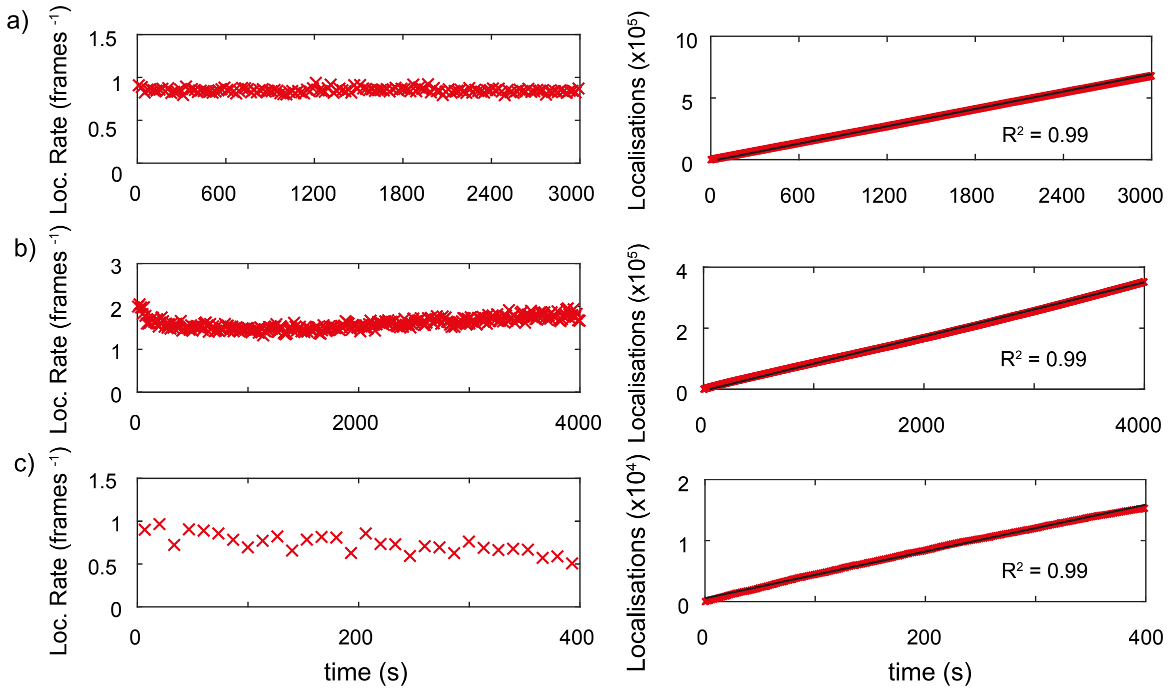

Supplementary Figure 4: resPAINT maintains a stable localisation rate over long durations indicative of a PAINT binding mode. a) WGA-PAJF$_{549}$ imaging of Jurkat T-cell membranes confirms that the localisation rate remains stable over 50 minute timeframes. **b)** WGA-HMSiR imaging of Jurkat T cell membranes similarly shows stable localisation over >1 hour timescales. **c)** anti-hCD45Fab imaging of a membrane protein is stable over 6 minutes.

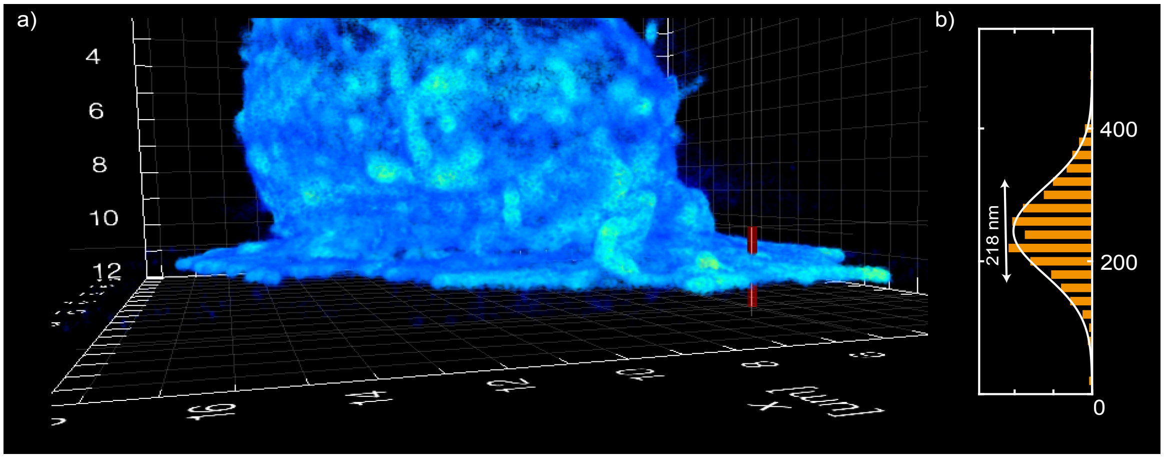

Supplementary Figure 5: Thickness of the adhered Jurkat T cell membrane measured with resPAINT. a) A line profile was applied to the skirt of the whole-cell image in Figure. 2g. **b)** A histogram of z position, shows the skirt FWHM thickness to be 218 nm.

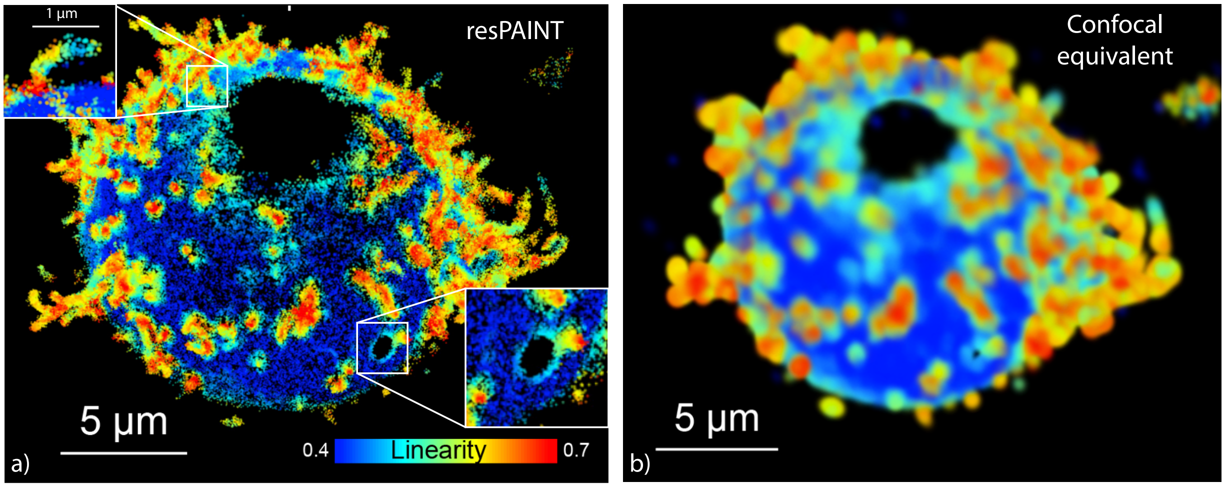

Supplementary Figure 6: Super-resolution imaging of cellular structures. a) DHPSF resPAINT imaging of a fixed Jurkat T cell, taken with WGA-HMSiR. Localisations are coloured by linearity, obtained using point cloud classification methodology.[14] The bottom right inset shows a distinct ~1 micron area where the membrane has formed a hole. The top left inset shows a clear microvilli (finger-like structure). **b)** The confocal resolution equivalent of (a) is shown by setting localisation precision to 250 nm in xy and 500 nm in z.

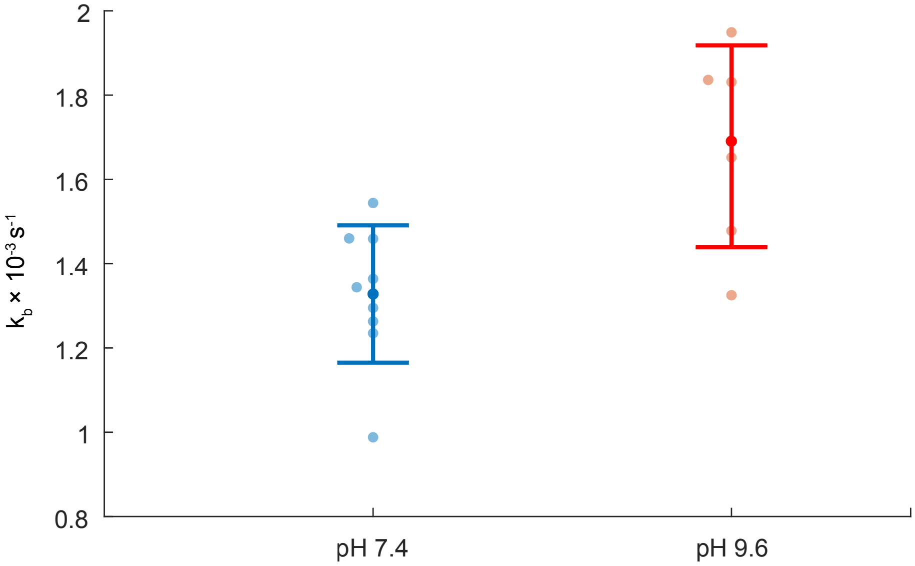

Supplementary Figure 7: Dissociation rate constants for anti-hCD45Fab-HMSiR on fixed cells. a) The dissociation rate constant, k_b, of the Fab fragment used in resPAINT of the membrane protein CD45 is measured on fixed cells by fitting the decay of fluorescence signal in a field of view to an exponential function at pH 7.4 and pH 9.6. The mean k_b was determined to be 1.33×10^{-3} s^{-1} at pH 7.4 and 1.68×10^{-3} s^{-1} at pH 9.6

a)

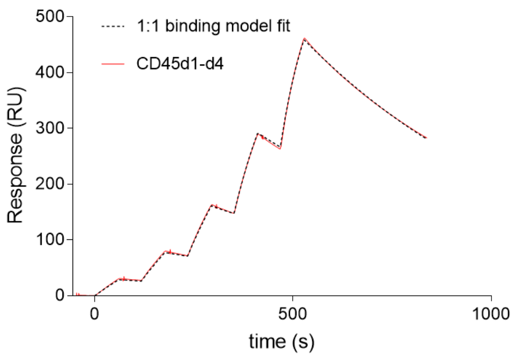

b)

Maximum concentration CD45d1-d4	k_a (M^{-1} s^{-1})	k_b (s^{-1})	K_D (nM)
250 nM	4.61e+4	1.59e-3	34.5
1000 nM	5.48e+4	1.89e-3	34.6

Supplementary Figure 8: Single cycle kinetic analysis of Gap8.3 binding to CD45d1-d4, using SPR at 20°C. a) representative plot of Gap8.3 binding two-fold serially diluted CD45d1-d4 with a maximum concentration of 250 nM (red) and 1:1 binding model fit (dotted black). b) Summary table of kinetic constants derived from 1:1 binding model, where the k_b was determined as 1.59×10^{-3} s^{-1}.

References

[1] R. A. Fernandes, K. A. Ganzinger, J. C. Tzou, P. Jönsson, S. F. Lee, M. Palayret, A. M. Santos, A. R. Carr, A. Ponjavic, V. T. Chang, C. Macleod, B. C. Lagerholm, A. E. Lindsay, O. Dushek, A. Tilevik, S. J. Davis, D. Klenerman, *Proc Natl Acad Sci USA* **2019**, *116*, 14002–14010.

[2] A. R. Carr, A. Ponjavic, S. Basu, J. McColl, A. M. Santos, S. Davis, E. D. Laue, D. Klenerman, S. F. Lee, *Biophys J* **2017**, *112*, 1444–1454.

[3] B. Diederich, P. Then, A. Jügler, R. Förster, R. Heintzmann, *PLoS ONE* **2019**, *14*, e0209827.

[4] L.-M. Needham, J. Weber, J. A. Varela, J. W. B. Fyfe, D. T. Do, C. K. Xu, L. Tutton, R. Cliffe, B. Keenlyside, D. Klenerman, C. M. Dobson, C. A. Hunter, K. H. Müller, K. O'Holleran, S. E. Bohndiek, T. N. Snaddon, S. F. Lee, *Chem. Sci.* **2020**, *11*, 4578–4583.

[5] M. Lew, M. D. Lew*, A. R. S. von Diezmann*, W. E. Moerner, *Protocol Exchange* **2013**, DOI 10.1038/protex.2013.026.

[6] A. Herbert, "GDSC ImageJ Plugins : Image J Analysis : ... : Sussex Centre for Genome Damage and Stability : Lifesci : Schools : Staff : University of Sussex," can be found under http://www.sussex.ac.uk/gdsc/intranet/microscopy/UserSupport/AnalysisProtocol/imagej/gdsc_plugins, **n.d.**

[7] A. Aristov, B. Lelandais, E. Rensen, C. Zimmer, *Nature Communications* **2018**, *9*, 2409.

[8] A. D. Edelstein, M. A. Tsuchida, N. Amodaj, H. Pinkard, R. D. Vale, N. Stuurman, *J Biol Methods* **2014**, *1*, e10.

[9] R. R. Sims, R. R. Sims, S. A. Rehman, M. O. Lenz, S. I. Benaissa, E. Bruggeman, A. Clark, E. W. Sanders, A. Ponjavic, A. Ponjavic, A. Ponjavic, L. Muresan, S. F. Lee, K. O'Holleran, *Optica, OPTICA* **2020**, *7*, 1065–1072.

[10] M. Ovesný, P. Křížek, J. Borkovec, Z. Švindrych, G. M. Hagen, *Bioinformatics* **2014**, *30*, 2389–2390.

[11] S. Bondza, E. Foy, J. Brooks, K. Andersson, J. Robinson, P. Richalet, J. Buijs, *Front. Immunol.* **2017**, *8*, 455.

[12] S. Wang, J. R. Moffitt, G. T. Dempsey, X. S. Xie, X. Zhuang, *Proc. Natl. Acad. Sci. U.S.A.* **2014**, *111*, 8452–8457.

[13] W. R. Legant, L. Shao, J. B. Grimm, T. A. Brown, D. E. Milkie, B. B. Avants, L. D. Lavis, E. Betzig, *Nat Methods* **2016**, *13*, 359–365.

[14] T. Hackel, J. D. Wegner, K. Schindler, in *2016 IEEE Conference on Computer Vision and Pattern Recognition (CVPR)*, **2016**, pp. 1610–1618.

Supplementary Movie 1: PAINT *vs.* resPAINT
We PAINT the apical surface of Jurkat T cell membranes using WGA, a lectin which binds to N-glycosyl moieties. The abundance of these targets renders WGA a popular membrane stain. For similar levels of background, resPAINT, with WGA-PAJF$_{549}$ (100 nM, photoactivation power density: 0.6 Wcm^{-2}), shows significantly increased localisation rate *vs.* conventional PAINT using WGA-AF$_{555}$ (100 pM). The exposure time is 30 ms.

Supplementary Movie 2: resPAINT optimisation
The effect of concentration and photoactivation power density is explored to determine optimal resPAINT conditions. The localisation rate increases with concentration and photoactivation power density, however so does the background levels. In the optimal condition (100 nM and 0.6 W cm^{-2} laser power - highlighted at 14 s, background < 92 photons pixel^{-1}) the localisation rate averages ~1.5 loc frame^{-1} at the apical cell surface. The exposure time is 30 ms.

Supplementary Movie 3: Whole-cell resPAINT imaging
Video representation of the whole Jurkat T cell membrane image from Figure 2c, highlighting the topographical features. Localisations are coloured by density as in Figure 2c. Grid spacing = 1 μm.

Supplementary Movie 4: Cell-surface interaction
Video representation of the whole Jurkat T cell membrane image from Figure 2g, highlighting the topographical features of the cell that are perturbed by electrostatic interactions with a PLL-coated glass surface. Localisations are coloured by depth as in Figure 2g. Grid spacing = 1 μm.

Supplementary Movie 5: resPAINT with HMSiR
Comparison of WGA-SiR, WGA-HMSiR at pH 7.4 and WGA-HMSiR at pH 9.6. Compared to SiR, HMSiR at pH 7.4 shows moderate localisation rate improvement. At the optimised pH of 9.6, the localisation rate is increased further, affording a 50-fold improvement compared to SiR. The exposure time is 20 ms.

Supplementary Movie 6: Long-term resPAINT
At an optimised probe concentration (0.1 nM), WGA-HMSiR imaging achieves a favorable localisation rate for DHPSF imaging (~1.77 loc. frame^{-1}). Three different timeframes are shown at the beginning (0 s), middle (2,000 s) and end (4,000 s) of a resPAINT experiment, demonstrating how the rate remains stable for at least 4,000 s. The exposure time is 20 ms.

Supplementary Movie 7: Optimal HMSiR pH
Comparison of WGA-HMSiR at pH 9.6 with WGA-HMSiR at pH 11.5. At the same concentration of probe, WGA-HMSiR at pH 11.5 shows fewer and dimmer puncta. Under the same laser power and exposure time (20 ms), the pH-dominated duty cycle is faster than the exposure time, thus reducing the amount of collected photons.

Supplementary Movie 8: Tetrapod PSF resPAINT
WGA-SiR compared to WGA-HMSiR in similar background conditions using the tetrapod PSF. WGA-SiR shows few localisations for the same level of background. Meanwhile, due to the large footprint of the PSF, there is significant overlap between localisations. The exposure time was 30 ms.

Supplementary Movie 9: Light field resPAINT
As for Supplementary Video 8, but with SMLFM. Similarly, due to the large DOF, there is significant overlap between localisations. Single emitters are split into nine perspective views, where a minimum of three views are required to fit a localization. The exposure time was 20 ms.

Supplementary Movie 10: Optimised Tetrapod PSF resPAINT
An example of WGA-HMSiR imaging of a Jurkat T-cell membrane with the tetrapod PSF. The PSFs do not significantly overlap at this concentration (1 nM), and the background is minimal, despite the 10 µm DOF. The exposure time was 20 ms.

Supplementary Movie 11: Optimised light field resPAINT
An example of WGA-HMSiR imaging of a Jurkat T-cell membrane with the SMLFM. The PSFs do not significantly overlap at this concentration (5 nM), and the background is minimal, despite the 10 µm DOF. The exposure time was 20 ms.

Supplementary Movie 12: resPAINT with a Fab
αCD45 Gap8.3 Fab-SiR imaging is compared to αCD45 Gap8.3 Fab-HMSiR imaging. With HMSiR, the localisation rate is feasible and allows imaging of the membrane protein CD45. A negative control experiment using murine T cells shows a significantly lower rate, corresponding to a chance coincidence of ~9 %. The exposure time was 20 ms.

Supplementary Movie 13: resPAINT simulation
Simulation of 2D PAINT experiment where WGA is binding to the cell membrane. For PAINT, 1 nM WGA is simulated for HILO and LLS excitation, while for resPAINT, 100 nM photoactivatable WGA (0.001 s^{-1} activation rate) is simulated. resPAINT greatly increases the localisation rate, while maintaining low background fluorescence.

Supplementary Movie 14: Fab resPAINT simulation
Simulation of 2D PAINT experiment where a Fab is binding to the cell membrane. For PAINT, 10 nM WGA is simulated for HILO and LLS excitation, while for resPAINT, 1000 nM photoactivatable WGA (0.001 s^{-1} activation rate) is simulated. resPAINT greatly increases the localisation rate, while maintaining low background fluorescence.
